# Rocking the world of innate immunity: an interview with Luke O'Neill

**DOI:** 10.1242/dmm.037838

**Published:** 2018-11-29

**Authors:** Luke A. J. O'Neill

## Abstract

Luke O'Neill is professor of biochemistry in the School of Biochemistry and Immunology at Trinity College Dublin, and his main research focus is innate immunity and its key output, inflammation. His work has explored the key sensors and mediators of infection, including the Toll-like receptors, inflammasomes and cytokines in the interleukin-1 (IL-1) family. Luke received the Royal Irish Academy Gold Medal for Life Sciences in 2012 and the European Federation of Immunology Societies Medal in 2014. He was elected a member of the European Molecular Biology Organisation (EMBO) in 2005 and a Fellow of the Royal Society in 2016. In this interview, Luke talks about the necessary collaboration between immunologists and cancer scientists, inflammaging, and his rock-star alter-ego as frontman of The Metabollix.


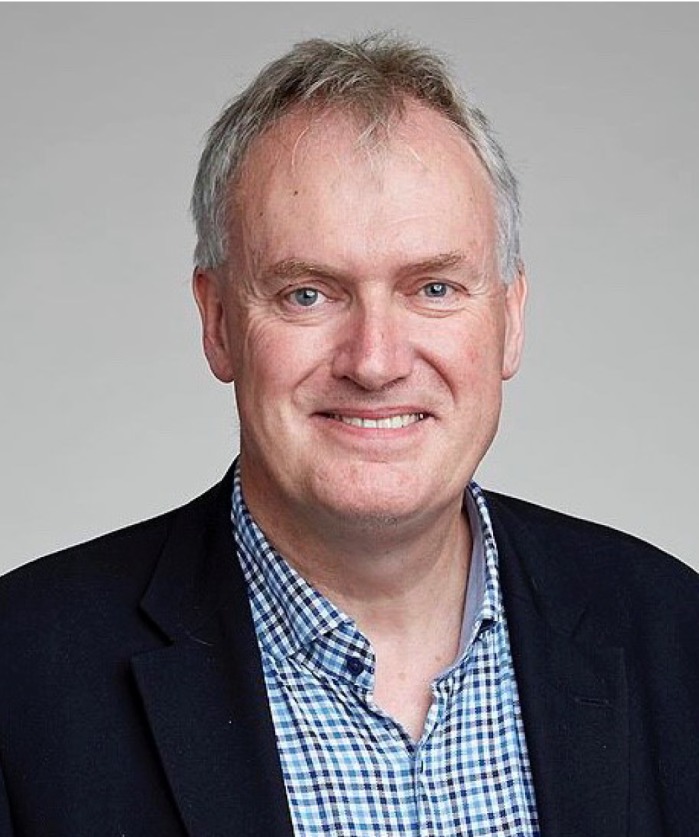


Luke O'Neill received his undergraduate degree in natural sciences (biochemistry) from Trinity College Dublin in 1985. Following his PhD in pharmacology from the University of London, he joined the Strangeways Research Laboratory in Cambridge as a postdoctoral researcher. He returned to Trinity College Dublin to set up his own laboratory to work on IL-1, the NLRP3 inflammasome and inflammation. Recently, his group has been focusing on the metabolic and circadian control of innate immunity. Luke published his popular science book, *Humanology*, in September 2018 to great acclaim.

**We typically start our interviews with a general question on your background. Have you always wanted to be a scientist?**

When I was about 11 or 12 I suddenly got interested in geology, of all things. I am from Ireland and I used to go hiking in the mountains and realised they were interesting. I liked science at school, had a very good teacher, including a good biology teacher. Even then, English was my favourite subject. Then, when I was in my final year before university, I realised that writing about Keats can get a bit boring after a while. Science was more attractive because I figured it was a better way to understand the world, as opposed to poetry.

**There is also more unknown than in studying the finite work of a single author, no matter how accomplished?**

I think there is a continuum between the arts and science. We are all trying to understand our place in the universe. You can do it through writing a poem or you can do it through studying DNA. And, being a scientist does allow a bit of creativity and imagination as well. What got me was data – you can do an experiment and then you can come to a conclusion based on that experiment.

**Do you still find that stimulation when you look at the data from your lab?**

PubMed is the answer [laughs]. Every day brings a new discovery in the fields of inflammation and immunology. It is a bit like trying to solve a complicated crossword puzzle with new clues coming along, and it is now beginning to fit together. Suddenly, things are a bit clearer and that is tremendously satisfying intellectually. Science is a great place to be if you are trying to crack complicated things.

**You've had a rather stable career, having done most of your research in Dublin. How, if at all, do you think being based at one institution has influenced your achievements?**

I began in Ireland, where I received my biochemistry degree, and then I went to the UK for my PhD postdoc. I was away for nearly 7 years when I got a job offer back in Ireland. I took it, thinking I might stay for a few years and then go somewhere else. But, when I began running my own lab, I liked being a PI, and work began to take off.

Science is international anyway. All my work is highly collaborative – there are authors from probably ten different institutions on one of our recent papers. So, back when I started, there was no need for me to move anywhere else. I could do great work in Dublin because of all these fantastic collaborators. I think that when you are young you should travel to get a different view of things. I did think about leaving a few times, but never did because I was never held back in Ireland and never felt I could not do my science there.

“I did think about leaving a few times, but never did because I was never held back in Ireland and never felt I could not do my science there.”

When I started my lab, immunology was a small field in Irish science, but it grew. Our school at Trinity now hosts perhaps 15 immunology groups. I have good colleagues and great students. I thought that recruiting good PhD students and postdocs might not be as easy as somewhere else, but my lab at present consists of seven nationalities in total: it is very diverse.

**Speaking of diversity, do you think there are lessons to learn from new cultures and trying to avoid cultural shock in science?**

Diversity is critical for bringing in new ideas to advance science. If we were all Irish guys in the lab doing experiments, that might not be effective. For some reason, mixing it up works. People bring a different perspective because they have different backgrounds. Gender balance is also massively important because men and women typically have different ways of solving problems. Having a diverse lab is also good fun when we convince people who are new to Ireland to drink Guinness, or when our Polish postdoc brings in vicious Polish vodka [laughs]. Ultimately, we are all on the same page as human beings and scientists.

**Inflammation is one of the hallmarks for cancer, and successful cancer immunotherapy approaches rely on concepts that you and your fellow immunologists helped uncover. As an immunologist yourself, do you think that there are any other lessons from immunology that the cancer community could learn from?**

Immunology has indeed come back into the frame for cancer researchers. Thirty or 40 years ago, immunologists knew the importance of harnessing the immune system, but other specialists weren't persuaded. I think that cancer research has changed recently with the renewed interest in metabolic changes in tumours – 100 years after the discovery of the Warburg effect. Immunologists then began to look at similar metabolic changes in the immune system, something that our group is also interested in.

The immunology world is informing the cancer world more and more, and that might mean new therapeutic approaches. This is the beauty of science: you fumble in the dark and then suddenly something interesting happens. I have worked on IL-1 for 30 years and now we know it is relevant in cancer, since blocking it seems to decrease the risk of lung cancer. This translational ‘proof in the pudding’ makes the areas of cancer and immunology more relevant to each other.

**Going back to the Warburg effect and its implications in the immune system: do you think that there are other lessons to be learned from cancer that could help answer open questions in innate immunity?**

When I began working as an immunologist, innate immunity was not seen as important. Immunology conferences almost exclusively discussed adaptive immunity. Then, suddenly, the innate world began to pick up with the discovery of Toll-like receptors. Your question is relevant because cancer cells release ligands for these innate receptors. That would mean that something had happened in the tumour to overproduce a ligand for the innate immune receptor. This can drive inflammation, which is pro-tumorigenic. But it also relates to other contexts, like autoimmune disorders, where ligands made by a non-transformed tissue drive the innate inflammatory response. There is no doubt that the cancer world can feed back into the inflammatory world by helping solve these problems. Remember that the immune checkpoint, a successful therapeutic target for cancer, was not discovered in cancer, it was first characterised in HIV infection.

We are inclined to live in our little worlds – cancer researchers focused on oncogenes for a long time, but those studies largely failed to deliver therapeutic breakthroughs. The immune checkpoint has given rise to breakthroughs. If we continue to study metabolism, we might eventually discover effective cancer therapies. Cancer was seen as a metabolic disease, a strange deviation in metabolism, before it was seen as a genetic disease. Now we know there is a crossover, because we learned that oncogenes drive this metabolic shift. The inflammatory process is not that different: if you think about very aggressive inflammatory tissue, say a rheumatoid joint, it looks and behaves like a tumour. It is not malignant, but it is as active as a tumour in burning up glucose, releasing ligands, disturbing the surrounding tissue. It equally hijacks the systems or pathways from other tissues to get an ecological benefit.

**Canakinumab, an IL-1 inhibitor, was recently shown to reduce the incidence of lung cancer in a clinical study that did not primarily focus on cancer. Could you tell us a bit more about the underlying biology and the wider implications?**

In this particular trial, started about 5 years ago, they built on evidence of IL-1's involvement in heart attacks. IL-1 recruits macrophages into the plaque, which exacerbates inflammation and causes changes in coagulation. This promotes cardiac events, so Paul Ridker and colleagues decided to explore whether IL-1 inhibition reduces the risk of a second heart attack. They found that canakinumab decreased the risk of secondary heart attacks by as much as 30% in a subset of participants. Heart attacks are partly inflammatory, and smokers have a higher risk. When treated with canakinumab, these patients, who were smokers, had a 67% decrease in risk of lung cancer incidence.

**Was that in comparison to placebo?**

Absolutely. These results feed into the idea that inflammation is actually a pro-tumour event – tumours like an inflammatory environment because it drives angiogenesis. Blocking IL-1 reduces inflammation. Interestingly, other studies showed that IL-1 was upregulated in lung cancer. So, the findings in this trial were unexpected, but not surprising. They will now have to repeat the trial because lung cancer risk was not a set objective.

**Do you think that, in the future, we will recommend anti-inflammatory agents to improve general health like vitamins, or would that be a double-edged sword?**

This is a tricky one, because you don't want to take pills if you don't have to. But there is this old poly-pill idea, where a person would take a mixture of different tablets to limit the effects of inflammation. So we might have a situation where people take these things prophylactically. The drug would have to be very safe, though, as toxicity is clearly a concern.

What is fascinating is that the diseases we are most interested in controlling are those of ageing. They relate to inflammaging: as you get older, your levels of inflammation increase, and that drives all these diseases.

**Inflammation indeed has a role in neurodegenerative diseases, for which ageing is the most important risk factor. Does this factor in with inflammaging?**

One theory is that noxious material builds up as you get older: uric acid crystals in gout, cholesterol in heart disease, β-amyloid in Alzheimer's, and α-synuclein in Parkinson's. These last two build up in the brain and the macrophages try to clear them. If this clearing is normal, the inflammation eventually resolves. In these diseases, however, too much build up causes chronic activation of macrophages, which becomes pathologic. NLRP3 seems to be the driver of that. We think that the ageing process might be a lacking capacity to clear these things for some reason.

**So it is not just that inflammation levels increase because we are getting older, it is the ability to clear the agents that cause inflammation that becomes less efficient?**

That is one idea, yes. If what I am saying is true, NLRP3 inhibitors could be an exciting prospect. We are exploring the idea of cranking macrophage activity down a bit to stop their over-activated state, in which they make too much IL-1. This drives the tissue injury that happens because of inflammation.

**The innate immune system, which is responsible for inflammation, is not something old and arcane – it is a smart machinery that evolves?**

The adaptiveness of the immune system as a whole blows my mind when I think about it. The fact that it undergoes gene rearrangements in anticipation of some kind of strange structure coming into your body – what a great discovery. What the pioneers in the field missed when studying adaptive immunity is that you need the innate system to drive this anticipation. Ralph Steinman's big contribution to this field was the discovery of dendritic cells, which present antigens.

Interestingly, innate immunity is much more complex than anyone ever imagined. We need to remember that most life on Earth only has innate immunity. It is clearly an ancient system, but very sophisticated. Immunologists made the mistake of thinking it was crude. But Mihai Netea discovered that the innate immune system has a sort of memory – it is known as trained immunity – and it responds more strongly when it re-encounters structures that it has seen before. So when you bolt on the idea of a memory mechanism to the innate immune system, it immediately becomes even more sophisticated.

**Speaking of ideas, you seem to be an inexhaustible source of them. Where do you find most of your inspiration?**

The mental smoothie – have you heard of that? You have to get your brain going, you read as much as you can, go to conferences, hear the talks. You watch TV that is relevant, maybe read Time magazine articles, talk to people…and then something happens in your brain and an idea pops out. Getting back to my first discussion about the arts: where do artists get ideas from? How do they write a new song or paint a picture? Again, there is something happening in their brains, absorbing from their environment and then turning it into something interesting. I think science is very similar. This mysterious process in our brains works its magic. The beauty is that, in science, you can test your idea – graduate students can anyway [laughs]! The best moments for me are when the lab is doing an experiment and I see a result that gets my mind going. I might have read a paper that is a bit different, a bit left field, and I think that links in with our results. When we are lucky, which doesn't always happen, this connection is right because the experiments behave themselves. My passion is inflammation and I want to know all about it. Whenever I hear anything on inflammation, I think about it and maybe come up with a new idea.

“Education is not about giving people a load of facts to learn, it is about drawing out the strengths of a person.”

**Do you incorporate this wisdom in your mentoring as well?**

When I take on someone in my own lab, the very first time I sit down with them I tell them I'm not expecting their experiments to work, and if the experiments are not working it doesn't mean they're failing. Managing expectations is the first thing. It's a great thrill when a PhD student begins to get interesting results. Watching them progress through is a wonderful thing. My job is to facilitate them. With postdocs, that is the luxurious time. Postdocs should be able to do things reasonably well and have a few years of real discovery. I tell them to enjoy this time and get on with the experiments.

When you get to the next stage and they have their own lab, they then realise it isn't quite so straight forward. I tell junior PIs to keep calm and just stick with it. Life is too short for people to be under terrible stress. We need to do better as a profession, and senior people must be cognisant of the stresses that postdocs and junior PIs have. You have to keep a sense of perspective on this. You have to publish all the papers because you don't know which one is going to count. So keep calm and keep at it. The biggest thrill for me initially is to be cited, because that means someone has read our work and is building on it. Then for their work to get cited is the next thrill because it means what we started is now extending. That's the dream, to have a pioneering role to play in a field. I also think giving talks is extremely important to get your work out there.

**Is this something you would advise people to do, especially more junior PIs?**

My advice to juniors is always say yes to a talk, because nobody reads properly anyway – most people read abstracts. If you give a talk, you can engage with the audience and they might remember what you talked about, which will have an impact on them, and they might change their experiments based on what they heard you say. The capacity to influence people through giving talks is important because that's the way to get your message out there.

**If you weren't a scientist, what would you be?**

What a terrifying question! I could only possibly be a scientist. I suppose looking back, it could have been a teacher or a librarian. Or a bookseller; something to do with education and the dissemination of knowledge. Education is not about giving people a load of facts to learn, it is about drawing out the strengths of a person. My other big passion is music. I am as enthusiastic about The Beatles as I am about innate immunity. I could easily teach kids about The Beatles. I also play a bit of music, so maybe I could have been a professional musician. Now that I am having my midlife crisis, I have a band – we are called The Metabollix. We are available for bookings!

“My other big passion is music. I am as enthusiastic about The Beatles as I am about innate immunity.”

**Do you leave Ireland – do you tour?**

Absolutely. We did a gig in Fiji and Australia. There is often a band at the closing dinner of every conference, so sometimes it's us. We get up and play. There was a big conference in Dublin in June 2017 and we put the band together. The conference was about immunometabolism, so we call ourselves The Metabollix. If I wasn't a scientist maybe I could be a full-time rock star with a band called The Metabollix.

